# Multidimensional EEG features integration with feature selection strategy for precision diagnosis of depressive disorders

**DOI:** 10.3389/fpsyt.2025.1624997

**Published:** 2026-01-12

**Authors:** Xiaodong Luo, Yanting Xu, Zihao Yan, Wei Liu, Bin Zhou, Gang Li, Yixia Zhu

**Affiliations:** 1 Psychiatry Department, The Second Hospital of Jinhua, Jinhua, China; 2 College of Engineering, Zhejiang Normal University, Jinhua, China; 3 College of Mathematical Medicine, Zhejiang Normal University, Jinhua, China

**Keywords:** depressive disorder (DD), electroencephalogram (EEG), feature selection, functional connectivity, machine learning

## Abstract

Depressive disorder (DD), a leading global cause of disability, lacks objective diagnostic biomarkers due to reliance on subjective clinical criteria. This study introduces an algorithm-driven framework integrating multidimensional EEG features, dynamic time-window optimization, feature selection and machine learning to address this gap. Resting-state EEG signals were acquired from 70 DD patients and 30 healthy controls (HC). Three-dimensional neurophysiological features, including power spectral density (PSD), sample entropy (SE), and phase lag index (PLI), were systematically extracted across variable time windows. The SVM-RFE algorithm eliminated redundant features, identifying an optimal subset that maximized classification accuracy through leave-one-subject-out cross-validation. Our model achieved exceptional classification accuracy of 94.48% using 10-second windows, outperforming conventional approaches. Critical biomarkers included beta rhythm alterations and cross-frequency functional connectivity patterns, demonstrating superior discriminative power for DD patients. The optimal feature subset emphasized the combined significance of spectral, nonlinear dynamic, and network-level characteristics in differentiating DD from HC. This framework establishes the first evidence-based integration of time-window and feature selection optimized multidimensional EEG features for DD identification, resolving key limitations in replicability and clinical translatability of existing methods. Beyond enabling high-precision objective diagnosis, the biomarker profile provides mechanistic insights into DD neuropathology, particularly beta rhythm dysregulation and aberrant cross-frequency coupling. These findings advance EEG-based precision psychiatry by offering a validated protocol for therapeutic monitoring and treatment personalization, bridging the critical gap between computational neuroscience and clinical practice in mood disorder management.

## 1 Introduction

Depressive disorder (DD) is a highly prevalent psychiatric disorder (1), characterized by persistent low mood, loss of interest, and decreased energy (2). It is widely regarded as one of the most debilitating psychiatric conditions (3). Over the past 30 years, the number of new cases worldwide has increased by approximately 50% (4). According to the World Health Organization (WHO), there were 280 million people globally suffering from DD in 2019 (5–7). Epidemiological studies in the United States found that the lifetime prevalence of major depressive disorder is approximately 16.6% (8). The incidence of suicide due to DD has risen and is now one of the top ten causes of death in the United States (9). DD not only exerts profound effects on both the physical and mental health of patients but also imposes a substantial economic burden on society, being the third leading cause of disability globally (10) and a major contributor to the global disease burden (11–13). Accurate and early diagnosis of DD is crucial for timely intervention and treatment, as well as for improving patient prognosis.

Although DD is a prevalent psychiatric disorder, its diagnostic accuracy and detection rate are greatly diminished. The current mainstream methods for diagnosing DD rely on clinical interviews and psychological assessments conducted by psychiatrists (14, 15). These assessments typically refer to the International Classification of Diseases, 10th edition (ICD-10) and the Diagnostic and Statistical Manual of Mental Disorders, 5th edition (DSM-5) (16). This process is strongly subjective and prone to misdiagnosis, with patients sometimes having to endure incorrect diagnoses and medications. Some patients only receive a final diagnosis after prolonged follow-up. Owing to the absence of objective laboratory diagnostic criteria, the accurate identification and diagnosis of DD remain suboptimal. Therefore, the development of objective diagnostic techniques for DD is of critical importance.

Electroencephalogram (EEG), a non-invasive neurophysiological technique (17), has emerged as a pivotal tool in identifying disease-specific biomarkers by capturing multidimensional electrophysiological signatures. Its integration with machine learning algorithms, leveraging high temporal resolution to decode complex neural dynamics, has revolutionized the diagnosis and management of neurological and psychiatric disorders (18, 19). Clinically validated applications span automated seizure detection (20), automatic sleep staging (21, 22), and prediction of various psychiatric disorders (23–25). In DD-related studies, EEG-based computational frameworks are being rigorously optimized across three critical domains: (1) early detection through discriminative feature extraction in prodromal stages, (2) individualized prediction of therapeutic responses to antidepressants or neuromodulation, and (3) dynamic prognostic evaluation via longitudinal biomarker monitoring (26). However, current classification accuracies remain constrained by the heterogeneous neurophysiological manifestations of DD and insufficient feature specificity. Recent advances emphasize the need to characterize nonlinear dynamical properties and cross-channel coupling patterns that may better reflect the disrupted neural homeostasis in DD.

Traditional EEG studies focus on analyzing linear features, including time domain, frequency domain (27, 28), and power spectral density (PSD) (29). Due to the nonlinear dynamic characteristics of EEG, linear features are insufficient to capture their complexity. Increasingly, nonlinear methods are being used to analyze DD (30), such as sample entropy (SE) to assess the complexity of EEG signal activity in patients with DD (31, 32), as well as multichannel mutual information(MI) (33), phase lag index (PLI) (34), and phase locking value (35), to explore functional connectivity and correlations between brain regions. Historically, studies predominantly utilized single features, such as the time-frequency power analysis (36), which may leads to a low DD classification accuracy (37). In recent years, multidimensional EEG features have been increasingly used for disease prediction, combining linear or nonlinear features in different ways to obtain new feature sets that often outperform the extraction of single features (38). This rationale is strongly supported by a growing body of evidence across various psychiatric disorders. Hosseinifard et al. used machine learning algorithms to combine linear and nonlinear features from 19-channel EEGs for DD classification, achieving an accuracy of 90% (39). Beyond DD, the synergistic effect of multidimensional EEG features has been demonstrated in Generalized Anxiety Disorder (GAD). For instance, Shen et al. integrated PSD, SE, and PLI, revealing that the combined feature set provided superior classification accuracy for GAD compared to any single feature type, highlighting the generalizability of this approach for capturing complex neuropathophysiology (40). Similarly, in schizophrenia research, the integration of spectral, complexity, and functional connectivity features has been shown to better differentiate patients from healthy controls by capturing complementary aspects of aberrant neural synchronization and dynamics (41, 42). Furthermore, studies on Alzheimer’s disease have successfully leveraged multidimensional EEG features to improve diagnostic precision (43). In summary, multidimensional EEG features are beneficial for improving the recognition rate of DD and represent a promising, biologically-informed framework for enhancing objective diagnostic accuracy across major psychiatric disorders.

Additionally, the optimization of temporal segmentation parameters represents a critical methodological consideration in EEG signal processing, as window length selection directly influences the trade-off between temporal resolution and feature stability in non-stationary neural signals. Contemporary approaches employ systematic windowing strategies to address the inherent non-stationarity of electrophysiological data while maintaining sufficient spectral resolution for clinical interpretation. Sorinas et al. demonstrated the operational significance of epoch duration through their comparative analysis of emotion classification performance, establishing 12-second windows as optimal for capturing sustained affective neural patterns while minimizing transient artifact interference (44). This finding aligns with the temporal characteristics of slow cortical potentials and cross-frequency coupling dynamics in emotional processing. Zhang et al. advanced this paradigm through their adaptive sliding-window framework, which incorporates spectral decomposition and dynamic time warping to optimize window parameters across distinct frequency bands (45). This spectral-adaptive approach reconciles the conflicting requirements of temporal precision in event-related potential detection and statistical reliability in power spectral density estimation. The empirical validation of these techniques underscores the necessity of context-dependent window optimization to balance signal stationarity assumptions with the temporal granularity required for clinically actionable biomarker discovery.

Based on the aforementioned findings, this study introduces a machine learning-based framework using time window optimization and multidimensional features fused to improve the diagnostic accuracy of DD. EEG signals were recorded from the DD patients and healthy controls (HC) during eyes-closed resting-state conditions. The signals were divided into five temporal window lengths: 4 s, 6 s, 8 s, 10 s, and 12 s. Three different EEG features, PSD, SE, and PLI were extracted. The leave-one-out method was utilized using a range of machine learning classifiers for classification. The three feature categories were assessed both independently and in combination to evaluate their classification performance. The Support Vector Machine - recursive feature elimination (SVM-RFE) algorithm was applied to identify an optimal feature subset with the highest classification accuracy. The selected multidimensional features were then classified using machine learning algorithms to optimize classification performance. Different from many existing studies that adopted subject-dependent cross-validation (Each subject has part of the samples in the training set and part of the samples in the test set), this study adopted strict subject-independent cross-validation, that is, leave-one-out method. This study aims to seek the key neurobiological markers of DD through the proposed analytical framework and to improve diagnostic accuracy for DD detection.

## 2 Materials and methods

### 2.1 Participants

The EEG data used in this study were collected from local hospital, and locally recruited individuals through professional screening. All participants in the experiment completed the Hamilton Depression Rating Scale - 17 (HAMD-17) before the formal data collection. All HC scored ≤7 on the HAMD-17, while patients with DD scored >17 on the same scale. All selected subjects were right-handed, with no other psychiatric disorders that could potentially impair brain function (such as dementia, schizophrenia, or anxiety disorders, except depressive disorder) and no physical impairments (such as severe cardiopulmonary, liver, kidney dysfunction, or autoimmune diseases). They had no history of drug or alcohol abuse and showed no signs of brain injury, with normal or corrected-to-normal vision. Each participant was prohibited from consuming alcohol and psychoactive drugs for 8 hours before the EEG recording. The EEG collection process occurred quietly without any other electromagnetic interference. The Ethics Committee of Zhejiang Normal University approved the study, and all participants provided written informed consent before participation.

**Figure 1:**
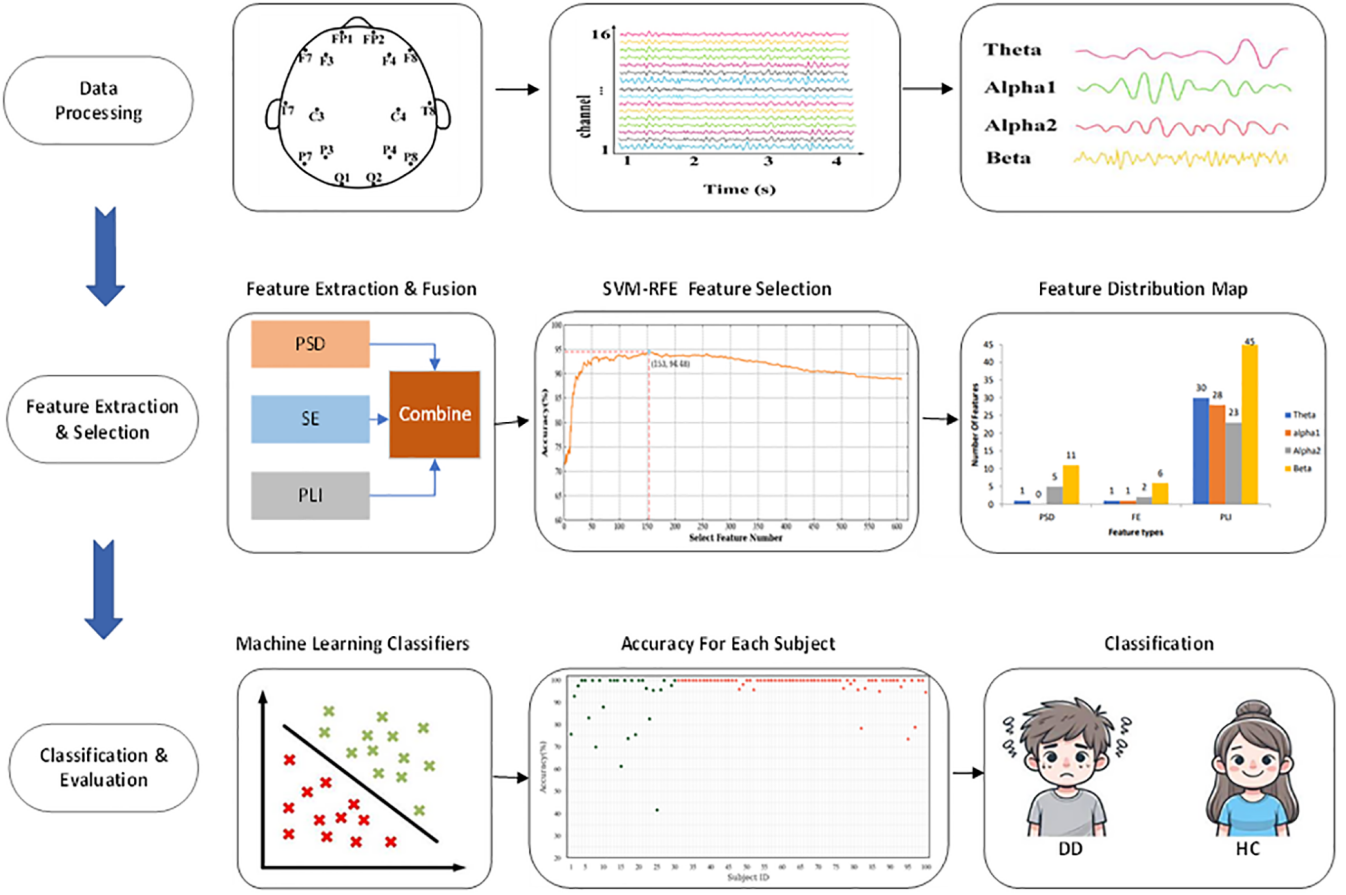
Overall research framework flowchart.

Based on the above conditions, this study collected data from 70 patients with DD and 30 HC. The age range of the DD patients was 19 to 61 years, with an average age of (53.34±15.04). All subjects had been ill for more than one month. The age range of the HC group was between 21 and 57 years, with an average age of (37.70±13.32). There was no statistical difference in age between the DD group and the HC group, but the HAMD-17 scores showed significant differences. The overall research framework of this study is illustrated in **Figure 1**.

### 2.2 EEG data acquisition and preprocessing

The EEG cap used in this study is Nicolet EEG TS215605. The electrodes are arranged according to the 10–20 international electrode system, retaining data from 16 EEG electrodes, as shown in **Figure 1**. It includes five brain interest regions: the frontal, temporal, parietal, occipital, and central regions. The experiment set the left and right earlobes (mastoid) as reference electrodes, with an EEG signal sampling frequency of 250Hz, ensuring that the impedance value of each electrode during the experiment is less than 5KΩ.

During EEG signal acquisition, it is very easy to be interfered with by various factors such as the environment, equipment, and physiology. These noises can negatively impact the signal quality. Preprocessing is a significant step in EEG data analysis, as it can effectively improve the signal-to-noise ratio, providing a reliable foundation for subsequent analysis and interpretation. The specific steps for preprocessing EEG in this study are as follows.

1. Filtering: Fourth-order Butterworth bandpass filter was used to remove unwanted frequency components from the raw signal, retaining the signal within a specific frequency range. A bandpass filter removes signals below or above a set cutoff frequency, keeping only the signals within that range. In this study, the range was set to 4–30 Hz, and the extracted EEG rhythms were theta (4–8 Hz), alpha1 (8–10 Hz), alpha2 (10–13 Hz), and beta (13–30 Hz).
2. Downsampling: Downsampling refers to reducing the sampling rate of high-sampling-rate EEG signals to a lower sampling rate, primarily to reduce data volume, improve computational efficiency, and decrease storage space. This study reduced the original EEG signal sampling rate from 250 Hz to 125 Hz.
3. Baseline Correction: The primary purpose of baseline correction is to eliminate the direct current offset generated by the recorded signal. When recording EEG signals, the signal may have varying degrees of direct current drift, affecting its accuracy and comparability. Baseline drift can remove the direct current offset in the signal, making the signal mean zero.
4. Artifacts Removal: Artifact removal aims to improve the signal-to-noise ratio, thereby better revealing the information expressed by the EEG signal. Common artifacts during the collection of EEG signals include muscle potential interference, ocular artifacts, and scalp induction artifacts. This study uses the method based on independent component analysis (ICA) to remove artifact signals. ICA can separate artifact signals from EEG signals and remove the artifact signals while preserving the information of the EEG signals.
5. Data Segmentation: Temporal window optimization was systematically implemented through parametric variation of epoch durations (4s, 6s, 8s, 10s, 12s) to balance stationarity assumptions with neural dynamic resolution, enabling comparative analysis of transient event-related potentials and sustained oscillatory patterns across frequency-specific neurophysiological processes. This multi-scale segmentation framework facilitates empirical identification of optimal temporal granularity for feature extraction in machine learning pipelines.

### 2.3 Feature extraction

Feature extraction is the most common data analysis method in EEG experiments, specifically summarized as single-channel, dual-channel, and multi-channel EEG analysis methods. The single-channel EEG analysis method is the most common EEG analysis method, including time domain analysis, frequency domain analysis, and nonlinear dynamics analysis. Dual-channel EEG analysis methods are commonly used to analyze the correlation characteristics of two-lead EEG signals, mainly to explore the brain functional connectivity characteristics between different leads and between different brain regions, and the standard analysis methods include MI, PLI, and coherence analysis. The multi-channel EEG analysis method refers to analyzing the functional state of a specific brain functional region, mainly based on the brain functional network analysis of complex network theory, including clustering coefficient analysis, characteristic path length analysis, and small world attribute analysis.

Based on literature research, this study will adopt a multidimensional feature perspective, using three widely used EEG features, including PSD features, SE features, and PLI features, encompassing linear, nonlinear, and brain functional connectivity analyses. These features have been repeatedly proven effective in detecting mental disorders. However, no one has yet compared and analyzed the advantages and dis-advantages of EEG characteristics in the DD group from a multidimensional feature perspective. The following will provide a detailed introduction to the specific calculation processes of the three features.

#### 2.3.1 PSD feature

PSD is a commonly used EEG signal processing method that decomposes EEG signals into the energy of different frequency components to reveal various brain activities. Existing research indicates that abnormalities in beta band power values are significantly related to anxiety symptoms, age, and other factors. The calculation process of PSD is as follows: Assuming the EEG time series signal is X(i). (*i* = 1, 2, 3, …, N), where N is the Nth sampling point of the EEG signal X(i), its spectrum can obtain through Fourier transformation, which can represent as X(f). Thus, its power spectrum $P_{x}\left(f\right)$ can be calculated using Equation 1. In Equation 2, h represents any EEG frequency band such as theta, alpha1, alpha2, and beta, with $f_{h}$ and $f_{l}$ denoting the lower and upper frequencies of the h band. For example, for the theta rhythm, $f_{h}$ is 4 Hz, and $f_{l}$ is 8 Hz.

(1) $$\;P_{x}\left(f\right)=\frac{1}{N}{\left|X\left(f\right)\right|}^{2}$$

(2) $$PSD\left(h\right)=\frac{1}{f_{h}-f_{l}}\int_{h}^{f_{l}}P_{x}\;df\;$$

This chapter uses 16 electrodes and four frequency bands. For EEG data with different time windows, a sample of 64 (16×4) PSD feature matrix will be obtained, where the sample represents each sample.

#### 2.3.2 SE feature

SE is a statistic that helps analyze time series data and has a wide range of applications in exploring data’s complexity, regularity, and randomness. The sample entropy value usually increases as the signal becomes more complex and random. The core idea of sample entropy is to measure the regularity and complexity of data based on patterns and recurrences in time series. Sample entropy is lower when similar patterns or subsequences occur frequently at multiple locations and higher when the patterns in the time series are relatively more random or irregular. Given a time series of length N, *X*={*x*(1), *x*(2),…, *x*(N)}, the procedure for calculating the entropy value of this sample is as follows:

1. Construct the time series X as an m-dimensional vector shown in Equation 3:

(3)  X(i)={x(i),x(i+1),…,x(i+m−1)},  i=1,2,…N−m+1

2 Define the distance between *X(i)* and *X(j)* as *d*[*X(i)*, *X(j)*] (*i* ≠ *j*), which is the maximum difference among their corresponding elements, as shown in Equation 4:

(4) $$d\left[X\left(i\right),X\left(j\right)\right]=\underset{k\in \left(0,m-1\right)}{\max}\left|x\left(i+k\right)-x\left(j+k\right)\right|$$

3. Given the threshold *r* (*r* > 0), count the number of instances where *d*[*X(i)*, *X(j)*]< *r* and compare it to the total number of vectors *N-m*, as shown in Equation 5:

(5) $$B_{i}^{m}\left(r\right)=\frac{1}{\text{N}-m}\text{num}\left\{d\left[X\left(i\right),X\left(j\right)\right]<r\right\}$$

4. Take the average of all the results obtained from as shown in Equations 5, 6:

(6) $$B^{m}\left(r\right)=\frac{1}{\text{N}-m+1}\overset{\text{N}-m+1}{\underset{i=1}{\sum }}B_{i}^{m}\left(r\right)$$

5. Then, increase the dimension m by one and repeat steps 1-4.

6. Theoretically, the sample entropy of this sequence is given in Equation 7:

(7) $$SampEn\left(m,r\right)=\underset{\text{N}\to \infty }{\lim}\left\{-\ln\left[\frac{B^{m+1}\left(r\right)}{B^{m}\left(r\right)}\right]\right\}$$

7. But in reality, N cannot be infinite; it must be a finite value. The estimated value of the sample entropy is given in Equation 8:

(8) $$SampEn\left(m,r,N\right)=-\ln\left[\frac{B^{m+1}\left(r\right)}{B^{m}\left(r\right)}\right]\;$$

In this study, we set *m* = 2 and *r* = 0.15×SD (standard deviation of the signal) to balance computational efficiency and physiological plausibility.

#### 2.3.3 PLI feature

PLI is an index used to estimate EEG phase coupling. It can accurately detect the asymmetry of phase differences. It can also describe the degree of phase synchronization between two channel time series signals, indicating brain functional connectivity. PLI is not significantly affected by volume conduction but is more noise-sensitive. The larger the PLI value, the higher the degree of phase synchronization between the two channel time series signals. The calculation process of PLI is as follows: Given the two-channel EEG time series signals *S_i_(t)* and *S_j_(t)*, first, calculate their instantaneous phase as shown in Equation 9. Next, the phase information for each time point can be obtained by calculating the magnitude and phase of the complex signals, and the phase difference can be obtained using Equation 10. Finally, the calculation of the PLI value can be obtained using Equation 11.

(9) $$\begin{array}{c} Z_{i}\left(t\right)=S_{i}\left(t\right)+jHT\left(S_{i}\left(t\right)\right) \end{array}$$

(10) $$\Delta \varphi (t)arg\left(\frac{z_{1}(t)*z_{2}(t)}{\left|z_{1}(t)\right|*\left|z_{2}(t)\right|}\right)$$

(11) $$PLI=\left|<sign\left(\Delta \varphi \left(t\right)\right)>\right|=\left|\frac{1}{N}\sum_{n=1}^{N}sign\left(\Delta \varphi \left(t\right)\right)\right|\;$$

$Z_{i}\left(t\right)$ is the EEG time series signal decomposed by the Hilbert Transform (HT), Δφ(t) is the phase difference between the two sets of time series signals, and *sign* is the sign function. For each EEG sample, feature extraction is performed using PLI, resulting in 16× (16-1)/2 = 120 feature values. A total of 4×120 = 480 PLI features can be obtained for four frequency bands.

### 2.4 Data augmentation

To address the pronounced class imbalance inherent in neuropsychiatric datasets (elevated DD: HC ratio), this study implemented Cross-Cluster Replication (CCR), a hybrid framework synergizing energy-based minority neighborhood purification with structured oversampling, to optimize model robustness against distributional skew. CCR’s dual-phase architecture first implements density-aware data purification to eliminate noisy majority-class encroachments in minority sample vicinities, followed by cluster-informed synthetic instance generation that preserves neurophysiological feature covariance. This approach strategically balances recall-precision tradeoffs critical in clinical diagnostics, where false-negative minimization is paramount yet must not catastrophically compromise specificity. The algorithm’s hierarchical resampling mechanism ensures minority class representation aligns with the neurodynamic complexity of depressive phenotypes while maintaining electrophysiological plausibility constraints, thereby enhancing classifier generalizability beyond conventional undersampling/oversampling paradigms. The specific calculation processes of CCR are as follows:

First, for dataset *X* containing the majority class and the minority class, set the oversampling proportion N and the nearest neighbor parameter *k*.

Second, randomly select a sample *X_i_* from the minority class samples and find the *k* nearest neighbors of this sample.

Further, for each minority class sample *X_i_*, repeat the following steps N times (randomly select a nearest-neighbor sample *X_nn_* from the *k* nearest-neighbors, generate a random number λ∈[0,1], and compute a new synthetic sample $X_{new}=X_{i}+\lambda \times X_{nn}-X_{i}$.

Finally, *X_new_* is added to the new dataset and combined with the original dataset.

### 2.5 Introduction to machine learning models

#### 2.5.1 Support vector machine

SVM was employed to find an optimal hyperplane for classifying multidimensional EEG features. Its capability to handle high-dimensional data and model complex, nonlinear relationships via kernel functions made it suitable for our EEG analysis.

#### 2.5.2 K nearest neighbors

KNN was used as a baseline model due to its simplicity and intuitiveness. It classifies samples based on the majority label among their K nearest neighbors in the feature space, providing a non-parametric comparison to more complex algorithms.

#### 2.5.3 Random forest

RF, an ensemble method based on decision trees, was selected for its robustness against overfitting and its ability to provide intrinsic feature importance rankings, which aided in interpreting the discriminative power of EEG features.

#### 2.5.4 Light gradient boosting machine

LightGBM was chosen for its high efficiency and speed when processing large-scale data. Its histogram-based algorithm accelerates training while maintaining high accuracy, making it suitable for exploring high-dimensional feature sets.

#### 2.5.5 Extreme gradient boosting

XGBoost was utilized as a highly optimized gradient-boosting framework known for its regularization techniques and superior performance in structured data tasks. It served as a strong benchmark for classification performance.

#### 2.5.6 Categorical boosting

CatBoost was included for its advanced handling of categorical features and gradient bias reduction, which ensures robust performance with minimal hyperparameter tuning, complementing our diverse model selection.

### 2.6 Validation strategy and hyperparameter optimization

To strictly prevent data leakage and ensure the generalization capability of the models, we adopted a Leave-One-Out Cross-Validation (LOOCV) framework. In each iteration of LOOCV, one subject was held out as the test set, while the remaining N−1 subjects constituted the training set. This process was repeated N times (where N=100) so that each subject served as the test sample exactly once.

Building upon this validation framework, to ensure reproducibility and fair comparison across different classifiers, we implemented a consistent hyperparameter optimization protocol (46, 47). Considering the high computational cost of the LOOCV scheme and the risk of overfitting associated with exhaustive searches on limited data, we employed a Targeted Grid Search strategy. For each classifier, the optimization was constrained to a focused parameter space centered on established heuristic baselines. The optimal configuration for each LOOCV iteration was selected based on the peak validation accuracy within the training fold. The specific hyperparameter search spaces, final standardized configurations, and methodological justifications for all employed classifiers (including SVM, Random Forest, XGBoost, etc.) are explicitly detailed in **Table 1**. This comprehensive summary ensures that the experimental conditions are fully transparent and reproducible.

**Table 1:** Hyperparameter search space and configuration for all classifiers.

Classifier	Key hyperparameters	Search space	Justification
SVM	Kernel Regularization ( C) Gamma ( γ)	‘rbf’ 1.0 ‘scale’	Standard for non-linear EEG features. Balanced penalty to prevent overfitting. Adaptive to feature variance ( $1/\left(n_{feat}\cdot \sigma^{2}\right)$).
KNN	n_neighbors ( k) Weights	5 ‘uniform’	Local neighborhood sensitivity check. Standard voting mechanism.
Random Forest	n_estimators Criterion	100 ‘gini’	Sufficient trees for stable variance reduction. Standard impurity measure.
XGBoost/ LightGBM/ CatBoost	n_estimators Learning Rate Max Depth	100 0.1 Default (Auto)	Balance between complexity and training time. Controls step size for gradient descent. Optimized internally by the algorithms.

SVM, Support Vector Machine; CatBoost, Categorical Boosting; XGBoost, eXtreme Gradient Boosting; LightGBM, Light Gradient Boosting Machine; KNN, K Nearest Neighbors.

The “Search Space” column indicates the specific values or standardized settings adopted in the final model to ensure deterministic reproducibility. For ensemble methods (XGBoost, LightGBM, CatBoost), parameters not listed were kept at their default values as provided by the Scikit-learn or respective library implementations.

### 2.7 Model evaluation metrics

To quantify classification performance, we utilized the confusion matrix (**Table 2**), which categorizes predictions into True Positives (TP), True Negatives (TN), False Positives (FP), and False Negatives (FN). TP and TN represent correctly classified positive and negative samples, respectively, while FP and FN denote misclassified instances. While Accuracy (calculated as shown in Equation 12) serves as the primary metric, we expanded the evaluation to include Recall, Precision, F1-score, and the Area Under the ROC Curve (AUC) to provide a robust assessment (calculated as shown in Equations 13–15). Given the LOOCV design where each test fold contains a single sample, these metrics were derived using a prediction pooling strategy, in which predictions from all subjects were aggregated to compute global performance measures. Furthermore, to quantify statistical uncertainty, the Accuracy is reported with its 95% Confidence Interval (Accuracy (%) [95% CI]), calculated based on the normal approximation of the binomial distribution.

**Table 2:** Confusion matrix.

Actual category predicted category	HC group	DD group
HC group	TP	FN
DD group	FP	TN

TP, True Positive; TN True Negative; FP, False Positive; FN, False Negative; DD, Depressive disorder; HC, healthy controls.

(12) $$Accuracy=\frac{TP+TN}{TP+TN+FP+FN}$$

(13) $$Precision=\frac{TP}{TP+FP}$$

(14) Recall=TPTP+FN

(15) $$F_{1}=\frac{2TP}{2TP+FP+FN}$$

### 2.8 Feature selection

This study addresses the challenge of high-dimensional neurophysiological feature analysis (n = 608 features encompassing PS, SE, and PLI), with 64 features derived from PSD (16 channels × 4 bands), 64 from SE (16 channels × 4 bands), and 480 from PLI (120 channel pairs × 4 bands), through a rigorous SVM-RFE framework optimized for differentiating DD (n = 70) from HC (n = 30). The methodology iteratively eliminates redundant features via backward selection, prioritizing features based on maximum margin weight magnitudes. To mitigate stochastic instability inherent in single-iteration rankings, the algorithm implements 100 iterations for the 100 subjects, generating probabilistic ranking matrices (100 × n), with final feature prioritization determined by modal frequency analysis across rank positions (The method for determining the final ranking results is to select the most frequently occurring feature from the first column of the 100 × n feature ranking results as the first feature in the final feature ranking and to select the two most common features from the first and second columns as the second feature, following this rule to determine the final feature ranking results). Optimal feature subset identification follows an incremental forward inclusion protocol, systematically evaluating classification performance with sequentially expanded feature combinations.

While emerging techniques such as graph neural networks and attention-based models have demonstrated strong capabilities in modeling complex and high-order feature interactions, their effective deployment in EEG-based psychiatric research demonstrably requires substantially larger datasets to mitigate overfitting and is often accompanied by significant computational burden and reduced interpretability (48–50). In contrast, the SVM-RFE framework adopted in this study provides a computationally efficient and highly interpretable feature-selection strategy that aligns well with the characteristics of moderate-sized clinical EEG datasets. Its linear kernel and explicit feature-ranking mechanism enable transparent identification of neurophysiologically plausible biomarkers, a requirement of particular importance in depression-related research where clinical interpretability and reproducibility remain central objectives. Furthermore, this approach offers a well-calibrated balance between dimensionality reduction and the retention of critical neurodynamic information, which is especially relevant given the markedly higher dimensionality of PLI connectivity features relative to PSD and SE. By distilling the multidimensional EEG space into a compact and clinically meaningful subset, the SVM-RFE framework ensures both analytical rigor and neurophysiological plausibility, thereby serving as a methodologically sound choice for the present investigation.

## 3 Results

### 3.1 Classification results for different classifiers

**Figure 2:**
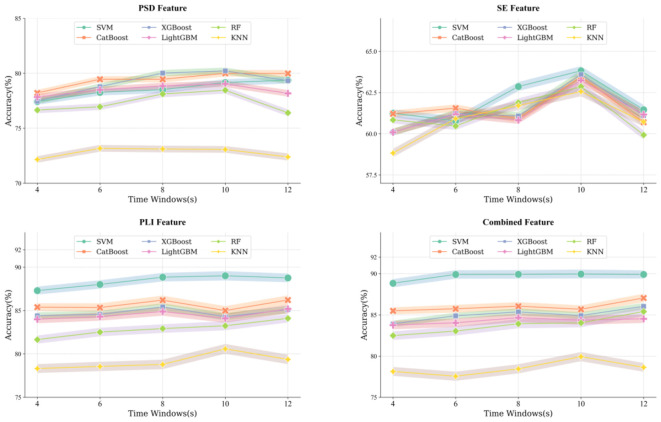
Classification results of all classifiers for each feature type under different time windows. PSD, power spectral density; SE, sample entropy; PLI, phase lag index; SVM, Support Vector Machine; CatBoost, Categorical Boosting; XGBoost, eXtreme Gradient Boosting; LightGBM, Light Gradient Boosting Machine; RF, Random Forest; KNN, K Nearest Neighbors.

This study employed a leave-one-out evaluation framework and multiple machine learning classifiers (SVM, CatBoost, XGBoost, LightGBM, RF, and KNN) to distinguish DD patients from HC. The classification accuracy of each individual feature type and their combination is shown in **Figure 2**, with the top-performing classifiers summarized in **Table 3**. XGBoost yielded the highest accuracy for PSD (80.33±29.77%), whereas SVM yielded the highest accuracy for SE (63.82±26.30%) and PLI (89.00±21.37%). When all three feature types were combined, SVM achieved an accuracy of 89.95±20.44%.

**Table 3:** Classification accuracy (Mean±SD) of top-performing classifiers (SVM) across different time window lengths.

Feature time	Combined (%)	PLI (%)	PSD (%)	SE (%)
4s	88.84±19.00	87.30±20.12	77.47±28.11	61.25±21.88
6s	89.90±18.72	88.01±20.44	78.79±28.53	60.78±22.88
8s	89.91±20.80	88.84±20.78	80.02±28.48	62.86±51.55
10s	89.95±20.44	89.00±21.37	80.22±29.21	63.82±26.30
12s	89.90±20.53	88.76±21.73	80.33±29.77	61.46±26.25

PSD, power spectral density; SE, sample entropy; PLI, phase lag index.

To provide a more comprehensive assessment of classifier performance, additional metrics—including Recall, Precision, F1-score, AUC, and Accuracy (%) with 95% Confidence Intervals—were calculated by aggregating predictions across all leave-one-out folds. As presented in **Table 4**, PLI exhibited the strongest single-feature performance (Recall 91.36%, Precision 93.01%, F1-score 92.18%, AUC 95.32%, Accuracy 89.00% [95% CI: 88.03–89.97%]). The combined-feature model further improved performance, achieving a Recall of 93.99%, Precision of 93.26%, F1-score of 93.63%, AUC of 96.49%, and the highest Accuracy at 90.90% (95% CI: 90.01–91.79%).

**Table 4:** Classification performance of top-performing classifiers (SVM) across different EEG feature categories.

Feature	Recall (%)	Precision (%)	F1_score (%)	AUC (%)	Accuracy (%) [95% CI]
PLI	91.36	93.01	92.18	95.32	89.00 (88.03–89.97)
PSD	81.48	89.25	85.19	86.61	80.00 (78.76–81.24)
SE	72.52	75.19	73.83	62.30	63.70 (62.21–65.19)
Combined	93.99	93.26	93.63	96.49	90.90 (90.01–91.79)

SVM, Support Vector Machine; PLI, phase lag index; PSD, power spectral density; SE, sample entropy; AUC, Area Under the ROC Curve; CI, Confidence Interval. Bold values indicate the best performance.

To evaluate the impact of the data augmentation strategy on handling class imbalance, this study conducted a comparative analysis between the proposed CCR method and the non-CCR dataset. As detailed in **Table 5**, the inclusion of CCR consistently enhanced classification performance across feature categories. Specifically, for the optimal combined feature set, accuracy improved from 85.01±30.37% (non-CCR) to 89.95%±20.44% (CCR), and PLI accuracy increased from 84.89±29.25% to 89.00±21.37%. These results confirm that CCR effectively mitigates majority class bias and is essential for the model’s robustness.

**Table 5:** Ablation study results comparing classification performance (Mean±SD) between the proposed CCR augmentation and the non-CCR imbalanced dataset across different feature categories.

Data strategy	Feature	Accuracy (%)
CCR	PLI	89.00±21.37
	PSD	80.22±29.21
	SE	63.82±26.30
	Combined	89.95±20.44
non-CCR	PLI	84.89±29.25
	PSD	75.80±37.53
	SE	70.54±35.83
	Combined	85.01±30.37

CCR, Cross-Cluster Replication; PLI, phase lag index; PSD, power spectral density; SE, sample entropy.

**Figure 3:**
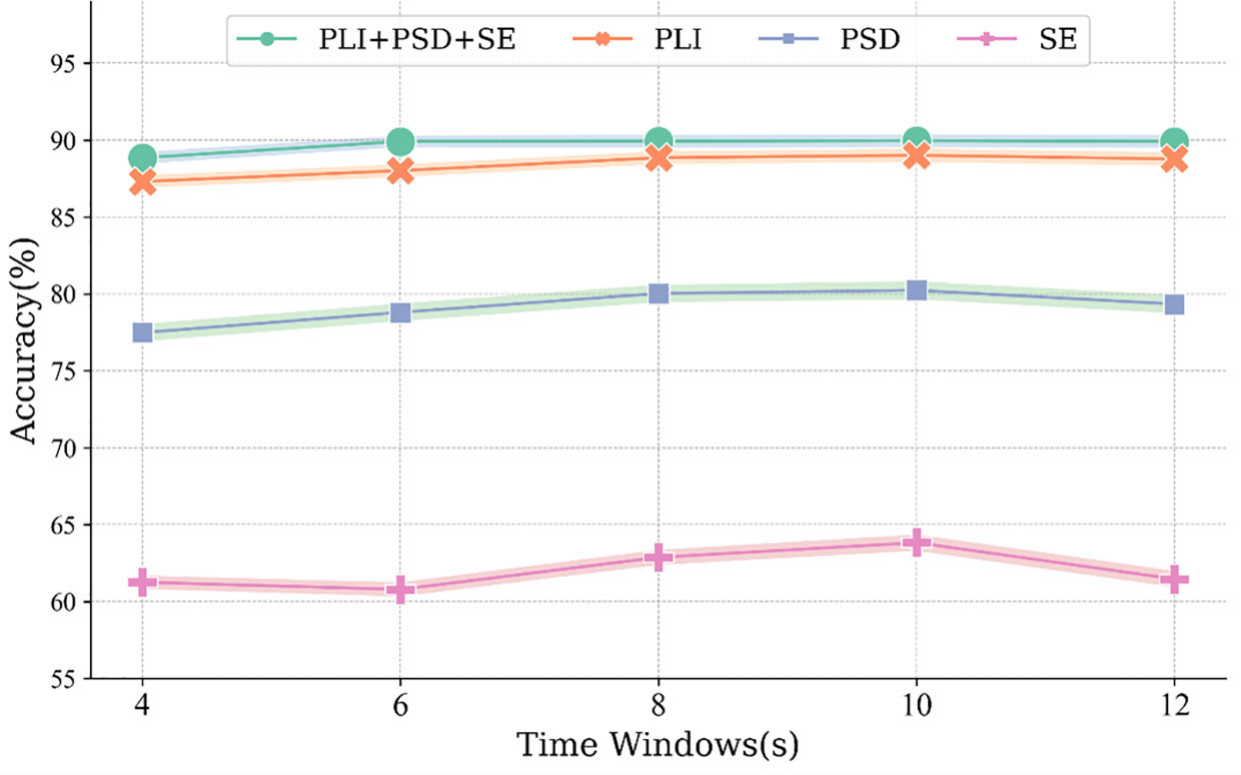
Classification effect of features under the best classifier SVM for different time windows. PSD, power spectral density; SE, sample entropy; PLI, phase lag index.

As shown in **Figure 3**, classification accuracy increased steadily from 4-s to 10-s windows, with the 10-s window yielding the highest performance under the SVM classifier. A subsequent decline was observed at the 12-s window.

### 3.2 Globally optimal sequence of features

**Figure 4:**
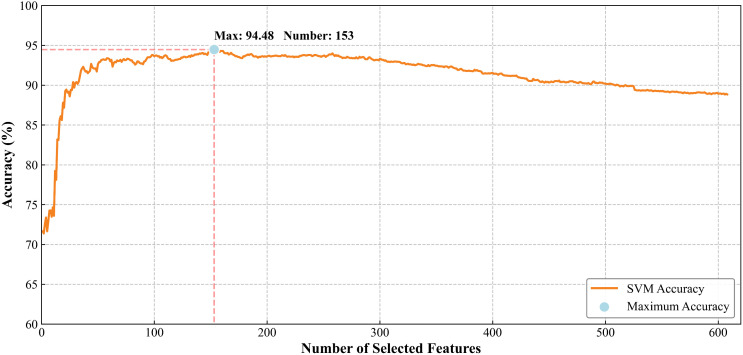
Classification result of the ranked feature subset. The blue dot means the highest classification accuracy. SVM, Support Vector Machine.

Given SVM’s excellent performance, we adopt the RFE feature selection method. Specifically, its feature ordering is computed for each subject. One hundred locally ranked features are obtained among 100 subjects, and the 100 locally ranked features are linearly summed to obtain the globally ranked feature sequence. Using the SVM classifier, features are added one by one for the global optimal feature sequence, and the results obtained are shown in **Figure 4**. Following feature selection, an optimal feature subset comprising 153 features achieved a classification accuracy of 94.48%. The detailed classification results for each feature within the optimal subset are presented in **Table 6**. Among the selected features, the subset included 17 PSD features, 10 SE features, and 126 PLI features. Notably, there were 11, 6, and 45 features corresponding to the beta frequency band, representing the largest proportion of features within each category.

**Table 6:** Distribution of features in the subset of optimal features.

Feature category	Rhythm	Number of features
PSD	Theta	1
	Alpha1	0
	Alpha2	5
	Beta	11
SE	Theta	1
	Alpha1	1
	Alpha2	2
	Beta	6
PLI	Theta	30
	Alpha1	28
	Alpha2	23
	Beta	45

PSD, power spectral density; SE, sample entropy; PLI, phase lag index.

**Figure 5:**
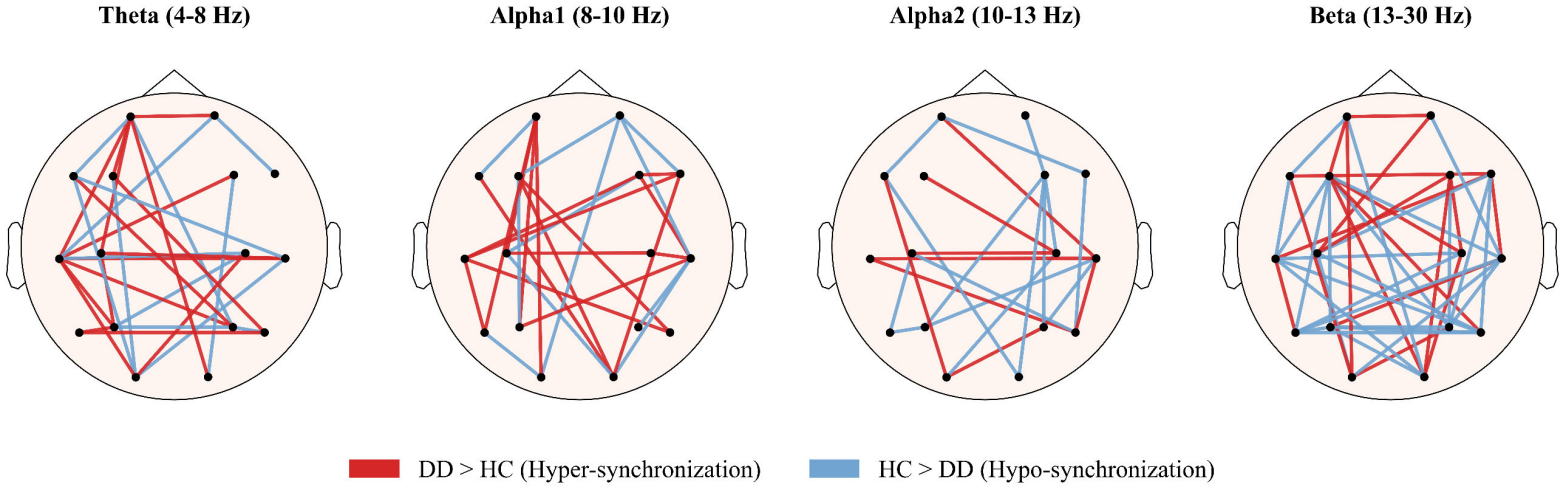
Topographical visualization of the discriminative PLI functional connectivity networks across four frequency bands. The nodes represent the 16 EEG electrodes. The edges represent the most discriminative functional connections selected by the SVM-RFE algorithm. red edges denote connections where DD patients exhibit significantly stronger synchronization than healthy controls, whereas blue edges denote weaker synchronization in patients. The spatial distribution highlights a dominant pattern of hyper-synchronization in the Beta band, reflecting abnormal functional integration in depression. DD, Depressive disorder; HC, Healthy controls.

To enhance interpretability, we visualized the discriminative PLI connections identified by the model across the four frequency bands (**Figure 5**). Red lines represent hyper-synchronization in DD relative to HC, whereas blue lines indicate hypo-synchronization. Theta and alpha1 bands showed stronger fronto-parietal and fronto-temporal synchronization in DD, while alpha2 and beta bands exhibited reduced connectivity primarily within centro-parietal and fronto-central regions. These patterns align with the distribution of high-ranking PLI features identified through SVM-RFE.

### 3.3 The accuracy of each test subject

**Figure 6:**
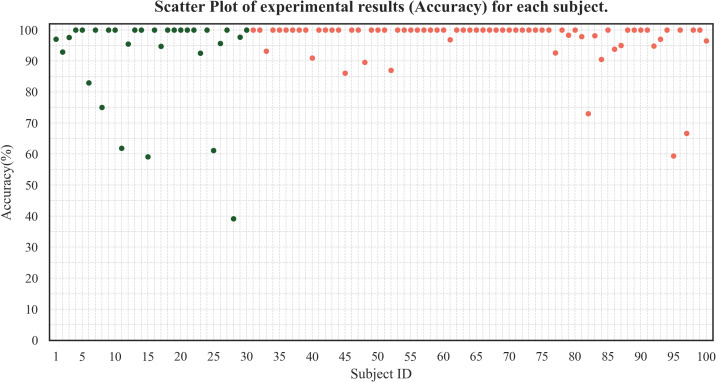
Scatter plot of subject-level classification accuracy. Each dot represents an individual subject (green: HC; red: DD). The distribution shows strong overall stability, with 92% of subjects achieving ≥80% accuracy and 99% achieving ≥50%. The observed variability is primarily driven by a single outlier, underscoring the robustness of the proposed framework for the vast majority of participants.

This study uses the optimal feature subset and SVM machine learning method to identify DD, resulting in the following scatter plot, as shown in **Figure 6**. The recognition accuracy for each individual ranged mainly between 80-100%, indicating stable performance. Excitingly, the number of subjects with an accuracy of over 80% was 92 (the total is 100), and the number of subjects with an accuracy of over 50% was 99 (the total is 100), indicating that the model has good predictability and stability.

## 4 Discussion

This study proposes a machine learning-based framework that combines time window optimization and multidimensional EEG feature selection to effectively construct a DD diagnosis model and improve the recognition rate of DD. The main conclusions are as follows: First, optimized 10-second EEG time window selection critically enhances DD identification. Second, multidimensional EEG feature fusion surpasses unimodal strategies, with PLI-driven functional connectivity disruption emerging as a neurophysiological signature of DD. Third, machine learning-aided feature subset optimization critically mitigates high-dimensional redundancy, enhancing model generalizability and translational robustness in computational psychiatry frameworks. The following sections will discuss these analysis results in more detail.

### 4.1 Temporal window length for stable EEG feature representation in depression diagnosis

This study indicates that a 10-second fixed-length window provides an optimal balance between achieving stable estimates of nonlinear complexity and connectivity features while preserving the quasi-stationary characteristics of EEG signals, thereby yielding robust classification performance in depression diagnosis. The choice of a fixed-window strategy—as opposed to adaptive or sliding-window approaches—was deliberate and grounded in several methodological and practical considerations. Historically, a wide range of time-window lengths has been applied in EEG signal processing (45, 51, 52), with fixed-length windows and sliding-mode windows, being among the most commonly used strategies (53). Fixed windows offer computational simplicity, standardized temporal resolution, and stable statistical conditions for estimating spectral and phase-based connectivity measures, which are essential for cross-subject comparability and the extraction of reproducible biomarkers in depression-related EEG research (54). They also minimize the risk of feature instability induced by variable overlap or step sizes in sliding windows. Sliding-mode windows introduce temporal overlap to improve continuity and sensitivity to gradual signal changes but may increase feature redundancy and attenuate low-frequency components (55). Although fixed windows may seem methodologically simpler than adaptive approaches, they are particularly well aligned with the objectives of depression-related EEG analysis, where standardized temporal resolution is crucial for cross-study comparability and the extraction of robust biomarkers. In the context of depression—where neural alterations often manifest as sustained oscillatory and network-level dysregulations—the controlled, parameter-invariant nature of fixed windows enables a clear assessment of how window duration per se influences feature discriminability. The controlled parameterization afforded by fixed windows enables a precise examination of how window duration influences multidimensional feature stability and classification performance, thereby establishing a reliable baseline for subsequent methodological innovation.

Substantial evidence from emotion recognition studies also indicates that affective neural states typically unfold over time spans exceeding 10 seconds (56–58), and similar considerations apply in automated EEG recognition of epileptic activity (59). From a computational perspective, longer windows improve efficiency by reducing segmentation frequency, whereas shorter windows better capture transient neural dynamics but impose higher computational demands.

Prior work consistently shows that window length critically shapes classification outcomes, underscoring the need for disorder-specific temporal optimization. For example, Luo et al. reported peak performance using a 10-second window for anxiety-related connectivity analysis (60), while Fang et al. identified 12-second windows as optimal for mixed anxiety–depression classification (61). However, their study did not delineate condition-specific optimal windows for DD and anxiety subgroups. Given the distinct biological, psychopathological, and genetic mechanisms underlying these disorders (1, 62, 63), generalized temporal parameterization across diagnostic categories remains methodologically unsound, necessitating disorder-specific optimization. Within the comparative framework of this study, the 10-second window demonstrated consistently robust classification performance across the tested resolutions, supporting its use as a practical reference scale for DD identification. This exploration of temporal parameterization not only contributes to the discussion on diagnostic precision but also provides novel methodological insights for temporal feature engineering in psychiatric EEG research. The observed variation in performance across window sizes highlights the conceptual importance of this parameter and warrants further investigation with larger cohorts to establish its definitive optimal value.

### 4.2 Synergistic integration of multidimensional EEG features enhances depression diagnosis

This study establishes that multidimensional integration of EEG features significantly enhances DD classification accuracy compared to single-dimensional approaches. By synergistically combining these complementary neurophysiological dimensions, our support vector machine (SVM) model achieved 89.95% accuracy, outperforming single-dimension benchmarks. This performance gain reflects the capacity of multidimensional features to capture distinct yet interrelated pathological mechanisms: PSD quantifies localized oscillatory abnormalities in cortical excitability, SE characterizes neural adaptability through signal complexity, and PLI maps interregional network dysregulation. Previous studies relying on isolated features, such as Li et al. (64) (PSD alone) and Avots et al. (65) (linear/nonlinear feature combinations), demonstrate the inherent limitations of single-dimensional analyses in addressing DD’s neurobiological complexity. Crucially, our findings align with emerging evidence that DD manifests as a multidimensional disorder requiring concurrent evaluation of rhythmopathology, complexity collapse, and network desynchronization (66).

Notably, PLI emerged as the most discriminative feature type (89.90% accuracy), consistent with prior findings that PLI-based connectivity dominates optimal biomarker sets in DD (67) and reflects aberrant phase synchronization patterns reported in clinical cohorts (68). The spatial distribution of key PLI connections in our results (particularly the pronounced frontal–cingulate and frontal–parietal interactions) further supports the notion that DD involves disrupted executive–limbic network coordination, aligning with evidence of impaired top-down regulation in depressive neuropathology (66). While PLI alone demonstrates strong diagnostic value, our multidimensional fusion framework achieved even higher performance (94.48%), corroborating studies showing that integrating PSD, SE, and PLI captures complementary aspects of oscillatory alteration, complexity reduction, and network-level dysregulation (40).

### 4.3 Machine learning combined feature selection leads to better depression diagnosis

Although this study used multidimensional features to construct the model, the overall accuracy was still not high before feature selection. We implemented SVM-RFE to eliminate low-weight features, with repeated leave-one-out cross-validation to identify the optimal feature subset, the optimal classification accuracy of 94.48% was achieved with 153 features. Notably, several studies have reported remarkably high classification accuracies of 99.08% (69) and 98.76% (70), which may be related to the different dataset partitioning methods used, such as subject crossover data division, which has a risk of data leakage, leading to an overestimation of the model’s actual performance. In contrast, the leave-one-out method can reduce such risks (71). In addition, feature selection and optimization are also commonly seen in the application of machine learning in other fields. There are many methods for feature selection, which tend to improve machine learning performance (72, 73). In the domain of DD diagnosis, Hierarchical Clustering and Spectral Network Fusion method significantly outperformed conventional feature selection approaches in classifying resting-state EEG signals for DD (74). Consistent with these findings, SVM-RFE method has been utilized to predict early treatment responses in DD, observing enhanced sensitivity compared to single-level prediction models (75). In summary, a review of the application of machine learning in the diagnosis and efficacy prediction of mental disorders (43, 76, 77), feature optimization is essential for improving the accuracy of machine learning. It can enhance model performance and increase classification accuracy. More efficient and reliable models can be constructed by employing reasonable feature selection. Our experimental results further validate that systematic feature selection constitutes an indispensable strategy for building efficient and reliable computational models.

Building on the optimized feature space, SVM-RFE revealed a feature subset dominated by Beta-band (13–30 Hz) activity, accounting for 64.7% of PSD, 60% of SE, and 35.7% of PLI features. This frequency-specific distribution suggests that abnormal Beta oscillations constitute a key electrophysiological signature of depressive disorders. This pattern is also consistent with findings from prior studies and advanced computational models (e.g., temporal–frequency attention), which frequently identify high-frequency hypersynchronization as an important discriminator in psychiatric conditions (48–50). The prominence of these Beta features points to specific pathophysiological mechanisms: the dominance of Beta-PSD reflects excessive local cortical excitability, while the prevalence of Beta-SE indicates rigid, low-complexity neural information processing. Collectively, these findings support the view that Beta-mediated cortical–limbic dysregulation constitutes a core electrophysiological signature of depressive pathology.

### 4.4 Limitations

The limitation of this method is that our current dataset comprises 100 subjects, including 70 with DD and 30 HC, and the sample size remains limited. Future studies should expand the dataset to enhance reliability for developing an effective DD diagnostic method. In addition, EEG data were acquired using a 16-channel clinical montage, which may limit spatial resolution compared with high-density (32–64 channel) systems. Although this configuration reflects real-world clinical practice and has been shown to capture stable large-scale spectral and connectivity patterns, future work should further validate the proposed framework using higher-density EEG recordings.

## 5 Conclusions

This study innovatively proposes a machine learning framework that integrates multidimensional EEG features, time window optimization, and feature selection for diagnosing DD. The results indicate that optimizing multidimensional EEG feature selection over a 10-second time window, along with global feature optimization, improves the recognition rate of DD using machine learning methods, providing a novel diagnostic model for DD. Furthermore, the predominance of PLI features in the optimal subset highlights aberrant phase synchronization as a key neurodynamic signature of DD, which offers strong guidance for the future development of EEG-based DD diagnostic models.

## Data Availability

The original contributions presented in the study are included in the article/supplementary material. Further inquiries can be directed to the corresponding authors.
